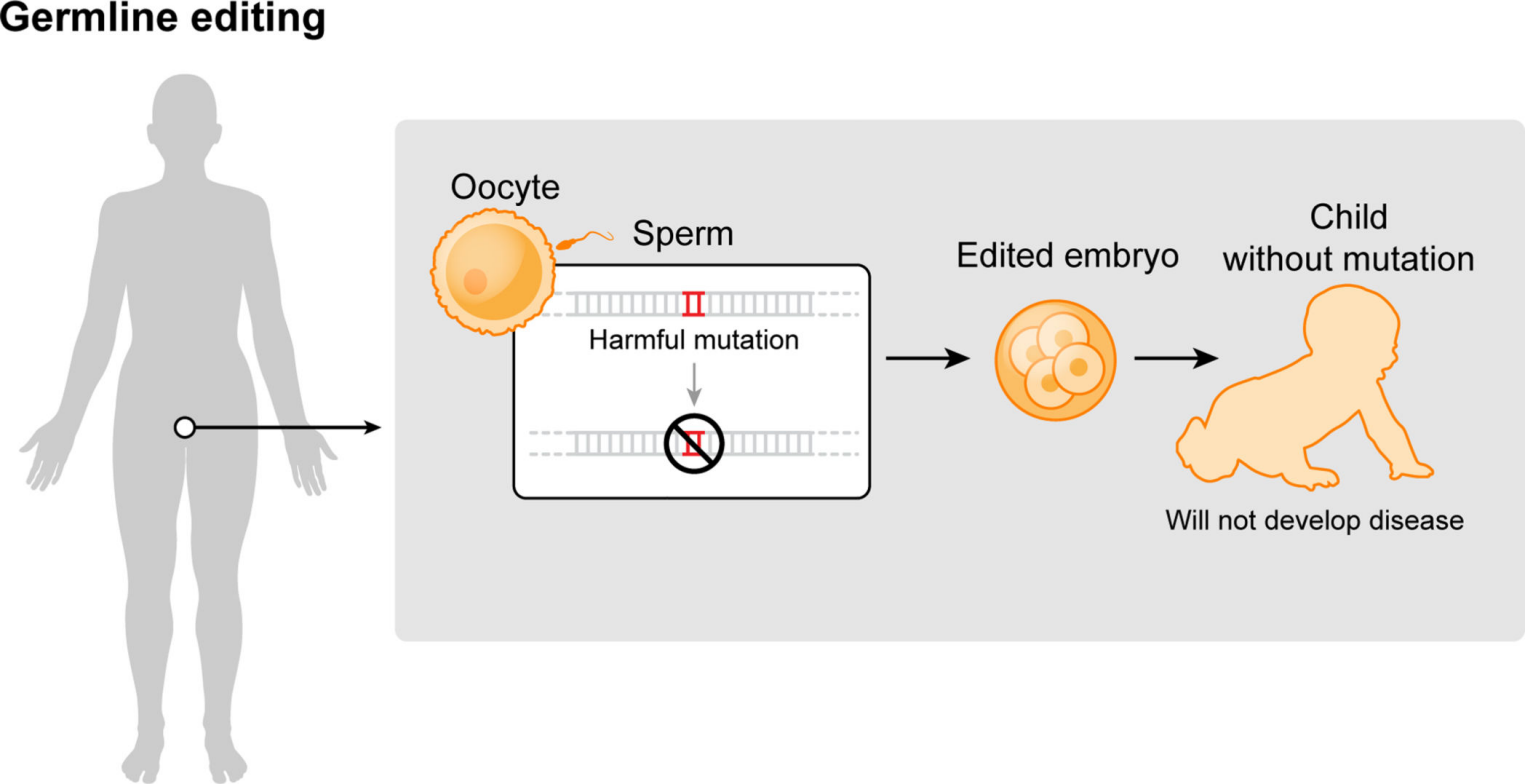# THE PROMISE AND CHALLENGE OF THERAPEUTIC GENOME EDITING

**DOI:** 10.1038/s41586-020-1978-5

**Published:** 2020-02-12

**Authors:** Jennifer A. Doudna

**Affiliations:** 1 Department of Molecular and Cell Biology, University of California, Berkeley, California 94720, USA.; 2 Department of Chemistry, University of California, Berkeley, California 94720, USA.; 3 California Institute for Quantitative Biosciences (QB3), University of California, Berkeley, California 94720, USA.; 4 Innovative Genomics Institute, University of California, Berkeley, California 94720, USA.; 5 Howard Hughes Medical Institute, University of California, Berkeley, California 94720, USA.; 6 MBIB Division, Lawrence Berkeley National Laboratory, Berkeley, California 94720, USA.; 7 Gladstone Institutes, University of California, San Francisco, California 94114, USA.

## Abstract

Genome editing, involving precise manipulation of cellular DNA sequences to alter cell fates and organism traits, offers the potential to both understand human genetics and cure genetic disease as never before. Scientific, technical and ethical aspects of employing CRISPR technology for therapeutic applications in humans are discussed, focusing on specific examples that highlight both opportunities and challenges. Genome editing is or will soon be in the clinic for several diseases, with more applications in the pipeline. The rapid pace of the field demands active efforts to ensure responsible use of this breakthrough technology to treat, cure and prevent genetic disease.

In the nearly seventy years since the discovery of the DNA double helix, technologies have advanced for determining, analyzing and altering genome sequences and gene expression patterns in cells and organisms. These molecular tools are the foundation of molecular biology, fueling the therapeutic industry by enabling profound advances in understanding the genetics of normal and disease traits. The ability to diagnose genetic diseases has developed rapidly with reductions in genome sequencing costs, extensive comparative analyses of human genome sequences and applications of high-throughput genomic screening. However, the dearth of therapies, much less cures, for genetic diseases has created a growing disconnect between diagnostics and treatments, underscoring the urgent need to develop therapeutic options. Mitigation or correction of disease-causing mutations is a tantalizing goal with tremendous potential to save and improve lives, representing a convergence of technical and medical advances that could eventually eradicate many genetic diseases.

Although methods for genome engineering and gene therapy have been of interest for decades, the development of engineered and programmable enzymes for DNA sequence manipulation has driven a biotechnological revolution^1–5^. In particular, fundamental research showing how clustered regularly interspaced short palindromic repeats (CRISPRs) and CRISPR-associated (Cas) proteins provide microbes with adaptive immunity has propelled transformative technological opportunities afforded by RNA-guided proteins. CRISPR-Cas9 and related enzymes have been used to manipulate the genomes of cultured cells, animals and plants, vastly accelerating the pace of fundamental research and enabling breakthroughs in agriculture and synthetic biology (reviewed in refs. 6–9). Building on past gene therapy efforts^10^, we are entering an era in which genome editing tools will be used to inactivate or correct disease-causing genes in patients, offering life-saving cures for people facing genetic disorders.

In this review I discuss therapeutic opportunities of genome editing, the ability to alter the DNA in cells and tissues in a site-specific manner. In addition to presenting current capabilities and limitations of the technology, I also describe what it will take to apply therapeutic genome editing in the real world. Comparison of somatic cell and germline editing highlights the importance of open public discussion about, and regulation of, this powerful technology.

## THE SCOPE OF GENOME EDITING APPLICATIONS

Although the genetics of human disease are often complex, some of the most common genetic disorders stem from mutations in a single gene. Cystic fibrosis, Huntington’s chorea, Duchenne muscular dystrophy and sickle cell anemia each represent diseases resulting from defects in just one gene in the human genome; on a global scale such monogenic diseases, of which ~5,000 are known, affect at least 250 million individuals. DNA sequencing in affected families has provided detailed information about the mutations that lead to each disorder, as well as correlations between specific genetic changes (genotype) and disease severity. These data in turn reveal DNA sequence alterations or corrections that could provide a genetic cure by either disrupting function of a toxic or inhibitory gene or restoring function of an essential gene.

Sickle cell disease and muscular dystrophy, two common human genetic disorders, provide instructive examples of diseases that could be treated or cured by genome editing in the foreseeable future. Sickle cell disease results from a single base pair change in DNA that in turn generates a defective protein with destructive consequences in red blood cells. Duchenne muscular dystrophy belongs to a set of muscle-wasting diseases resulting from DNA sequence changes that disrupt normal production of a protein required for muscle strength and stability. A closer look at each of these diseases illustrates the ways that genome editing could offer therapeutic benefit to patients.

**Fig. 1:**
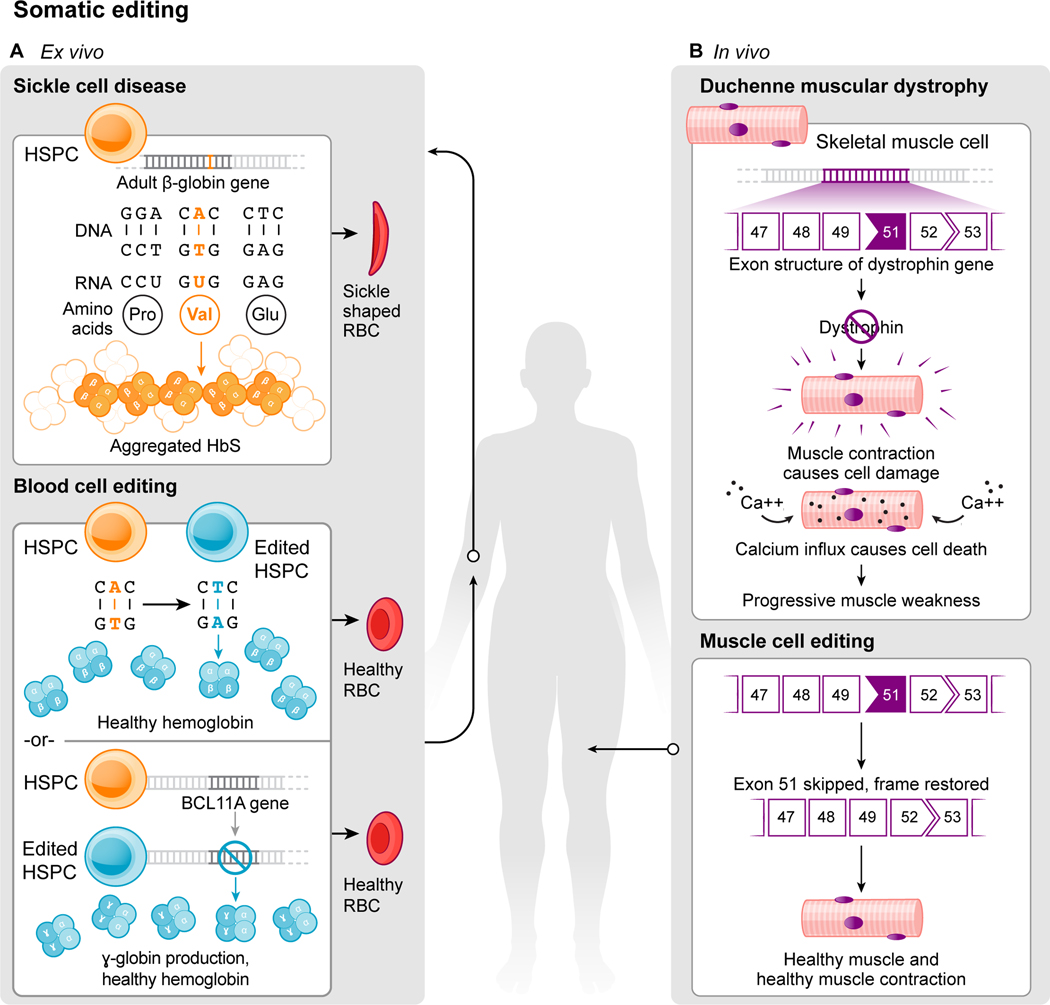


Sickle cell disease occurs in people that have two defective copies of the gene encoding β-globin, the protein required to form oxygen-carrying hemoglobin in adult blood cells. Described originally by Linus Pauling and colleagues^11^ and mapped to a genetic locus in the 1950s^12^, a single A to T mutation results in a glutamate-to-valine substitution in β-globin (Fig. 1). This seemingly small change causes the defective protein to form chain-like polymers of hemoglobin, inducing red blood cells to assume a sickled shape that leads to occluded blood vessels, pain and life-threatening organ failure. Although bone marrow transplantation can cure the disease, it requires using cells from an individual whose immune profile matches that of the patient. In principle, sickle cell disease could be cured by removing blood stem cells – hematopoietic progenitors – from a patient and using genome editing to either correct the disease-causing mutation in β-globin or activate expression of ɣ-globin, a fetal form of hemoglobin that could substitute for defective β-globin (Fig. 1). The edited stem cells could then be transplanted back into the patient, where their progeny would produce normal red blood cells.

The ability to conduct the editing in cells extracted from sickle cell patients makes their disease – and other blood disorders – some of the more tractable pathologies that could be treated by genome editing in the near term. Most genetic diseases, however, will require genome editing of cells in the body (*in situ*) to correct a genetic defect associated with disease. Muscular dystrophy exemplifies this type of disorder because it involves weakening and disruption of skeletal muscles over time (reviewed in refs. 13,14). The most common type, Duchenne muscular dystrophy (DMD), affects one in 5,000 males at birth who inherit mutations in the gene encoding dystrophin, a scaffolding protein that maintains the integrity of striated muscles (Fig. 1). Over time these patients lose the ability to walk and eventually succumb to respiratory and heart failure, typically causing death by the third decade of life. In contrast with therapies to delay disease progression, genome editing offers the possibility of permanent restoration of the missing dystrophin protein. Although >3000 different mutations can cause DMD, most occur at hotspots within the dystrophin gene. Notably, restoration of a small percentage (~15%) of normal dystrophin expression levels can provide a clinical benefit^15^.

To treat or cure monogenetic disorders like sickle cell disease and DMD, it will be important to match the underlying genetic defect with the best genome editing approach. In each case this involves multiple considerations including the type of editing needed, the mode of cell or tissue delivery required and the extent of gene knockout or correction that will provide therapeutic value.

The next section describes current genome editing technologies that offer the potential of curative human genome editing.

## GENOME EDITING STRATEGIES

**Fig. 2:**
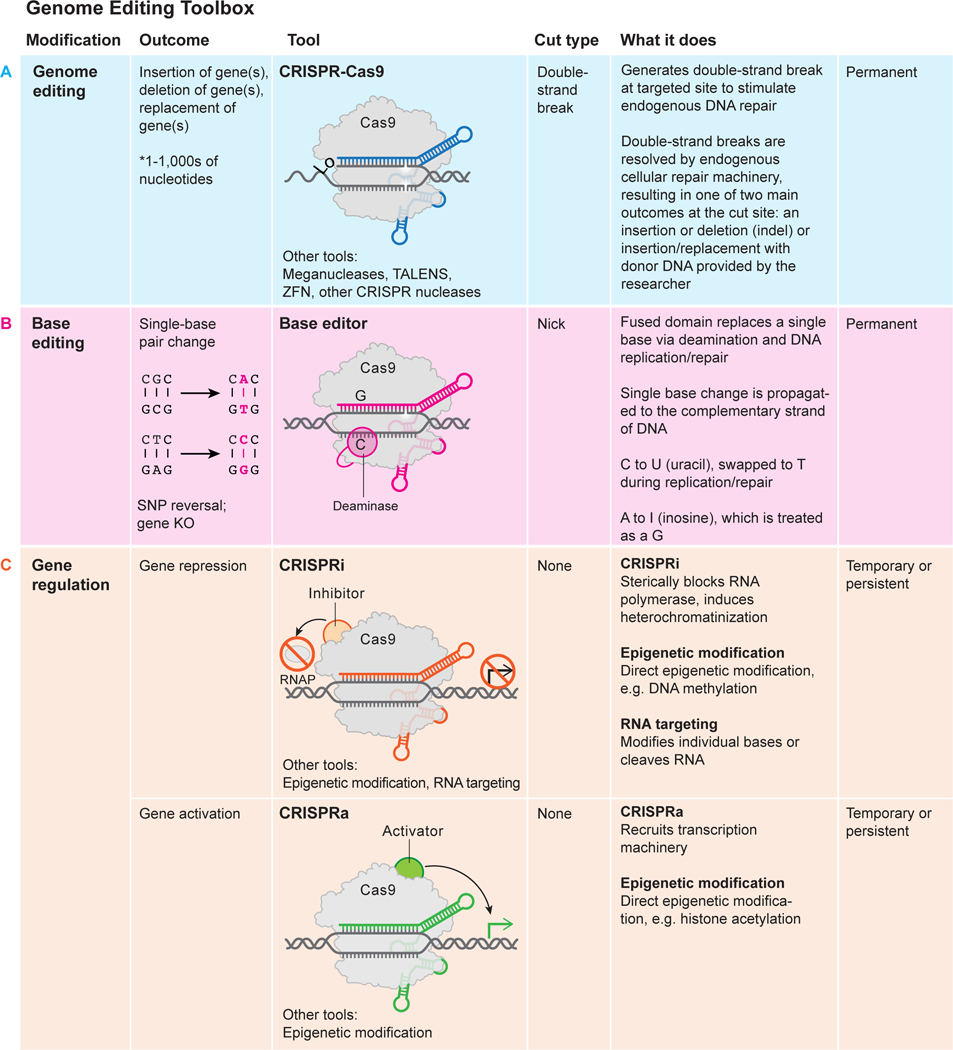


Engineered DNA-cleaving enzymes including zinc-finger nucleases (ZFNs) and transcription activator-like effector nucleases (TALENs) demonstrated the promise of therapeutic genome editing. These early technologies enabled inactivation of the HIV co-receptor gene CCR5 in somatic cells^16^, mitigation of the globin gene mutation in hematopoietic stem cells^17,18^ and engineering of immune system cells to treat childhood cancer^19^. To realize this promise, the development of CRISPR-Cas9 for genome editing offers a simpler technology that has been adopted widely due to the ease of programming its DNA binding and modifying capabilities. Cas9 is a protein that assembles with guide RNA, either as separate crRNA and tracrRNA components or a chimeric single-guide RNA (sgRNA), to create a molecular entity capable of binding and cutting DNA^1^. Importantly, DNA binding occurs at a 20-base pair DNA sequence that is complementary to a 20-nucleotide sequence in the guide RNA and can be readily altered by the experimenter^1,20^ (Fig. 2). The DNA recognition site must be adjacent to a short motif (protospacer adjacent motif, PAM) which acts as a switch, triggering Cas9 to make a double-stranded DNA break within the targeted sequence^1,20^. In cells of all multicellular organisms, including humans, such double-stranded DNA breaks induce DNA repair by endogenous cellular pathways that can introduce alterations to the DNA sequence, including small sequence changes or genetic insertions^21,22^. Although CRISPR-Cas9-induced genome editing is effective in virtually all cell types, controlling the exact editing outcome remains a challenge in the field, as discussed later in this review.

Although *S. pyogenes* (SpCas9) is the CRISPR-Cas enzyme most commonly used for genome editing and genetic manipulation, a growing collection of natural and engineered Cas9 homologs and other CRISPR-Cas RNA-guided enzymes is expanding the genome manipulation toolbox^6,23,24^. It is the intrinsic programmability present in this diversity of enzymes that underscores the utility of CRISPR-Cas technology for genome editing and other applications including gene regulation and diagnostics (Fig. 2).

For safe and effective clinical use *ex vivo* and *in vivo*, genome editing needs to be accurate, efficient and deliverable to desired cells or tissues. CRISPR-Cas9-induced DNA cleavage induces genome editing during double-strand DNA break repair by non-homologous end joining and/or homology-directed repair (Fig. 2). Homology-directed repair, requiring the presence of a DNA template, is in most cases used by the cell less frequently than non-homologous end joining. Furthermore, both types of repair can happen in the same cell, creating different alleles of an edited gene. Two concurrent double-strand DNA breaks can induce chromosomal translocations. For these reasons, an active area of CRISPR-Cas technology development involves controlling DNA repair outcomes to ensure that the desired genetic change is introduced.

**Fig. 3:**
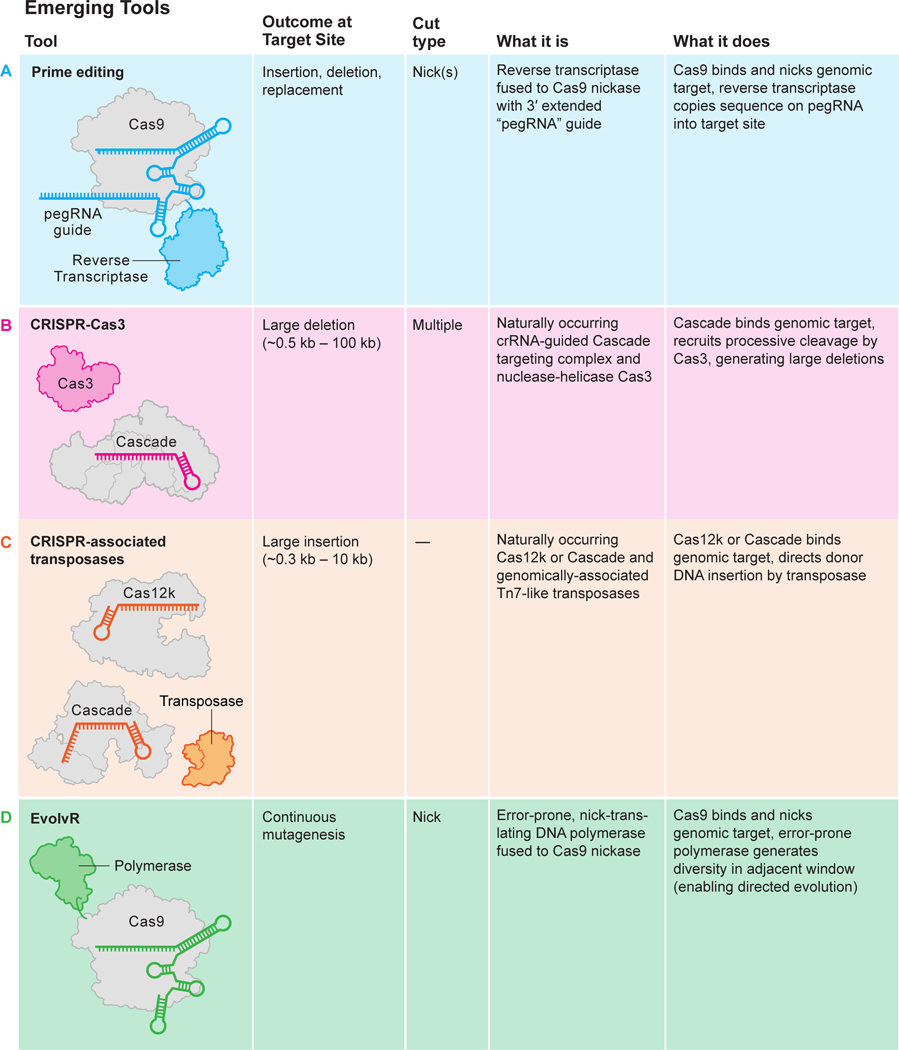


Alternatives to DNA cleavage-induced editing include using CRISPR-Cas9 for direct chemical sequence alteration (base editing)^25,26^, providing RNA templates for gene alteration (prime editing)^27,28^, and for transcriptional control (CRISPR interference, CRISPRi; CRISPR activation, CRISPRa)^29,30^ (Fig. 3). In addition, it may be possible to control gene outputs through Cas9-mediated epigenetic modification (reviewed in refs. 31, 32). While these methods have been used in cultured cells, they are not yet ready for clinical use until matters of specificity^33,34^ and delivery are addressed.

Two strategies to mitigate or cure sickle cell disease take advantage of demonstrated strategies for site-specific genome editing (Figs. 1, 2). The first involves restoration of the normal β-globin gene sequence by homology-directed repair^35^. The second approach is to activate expression of ɣ-globin, the fetal form of hemoglobin typically silenced in adult cells, by disrupting ɣ-globin repressors^36–41^ or their binding sites in the ɣ-globin gene promoter^40,42,43^. These genome-editing strategies require harvesting a patient’s hematopoietic progenitor/stem cells (HPSCs), either to correct the β-globin mutation or to restart expression of ɣ-globin, and then re-introducing the edited cells into the bone marrow. Major progress in delivering to^44^ and handling HPSCs has resulted in formidable efficiencies of mutation correction or mitigation^18,45–47^ that are expected to be curative.

Such an approach, while requiring bone marrow transplantation, would remove the need for a compatible bone-marrow donor and thus provide a path for treating and potentially curing many more people than can be treated at present. As discussed below, improvements in *in vivo* delivery technology may one day enable treatment without requiring bone marrow transplantation, which would reduce both expense and patient hardship.

While *in vivo* editing may resolve some of the issues with *ex vivo* sickle cell therapies, studies in muscular dystrophy illustrate that other challenges arise when attempting *in situ* gene correction. Three reports highlight both the tremendous potential and the significant remaining challenges to using genome editing to treat or cure muscular dystrophy in humans. In the first study, a DMD mouse model was created using CRISPR-Cas9 to generate a common deletion (ΔEx50) in the dystrophin gene that occurs in DMD patients^48^. The severe muscle dysfunction in the ΔEx50 mice was corrected by systemic delivery of adeno-associated virus (AAV) encoding CRISPR-Cas9 genome editing components, restoring up to 90% of dystrophin protein expression throughout skeletal muscles and the heart of ΔEx50 mice. The second study used CRISPR-Cas9-mediated genome editing to remove a mutation in exon 23 in the *mdx* mouse model of DMD, providing partial recovery of functional dystrophin protein in skeletal myofibers and cardiac muscle^25,26,49^. In the third study, dogs harboring the ΔEx50 mutation corresponding to a mutational “hotspot” in the human *DMD* gene were treated using CRISPR-Cas9^50^. After virus-mediated systemic delivery in skeletal muscle, dystrophin levels were restored to 3–90% of normal, and the muscle tissue appearance in treated dogs was improved. Although promising, these reports, as well as early-stage data from patients treated with *in vivo* gene editing using ZFNs, highlight the gap between animal studies and applications in humans^51–53^ and underscore the need for improved methods for *in situ* delivery, as discussed in the next section. An early stage clinical trial using *in vivo* CRISPR-Cas9 delivery to the eye to treat congenital blindness^54^ and a close-to-the-clinic program for liver gene editing^55^ will shortly provide key first-in-human data to inform the direction of that effort.

## TOWARDS TISSUE-SPECIFIC DELIVERY

**Fig. 4:**
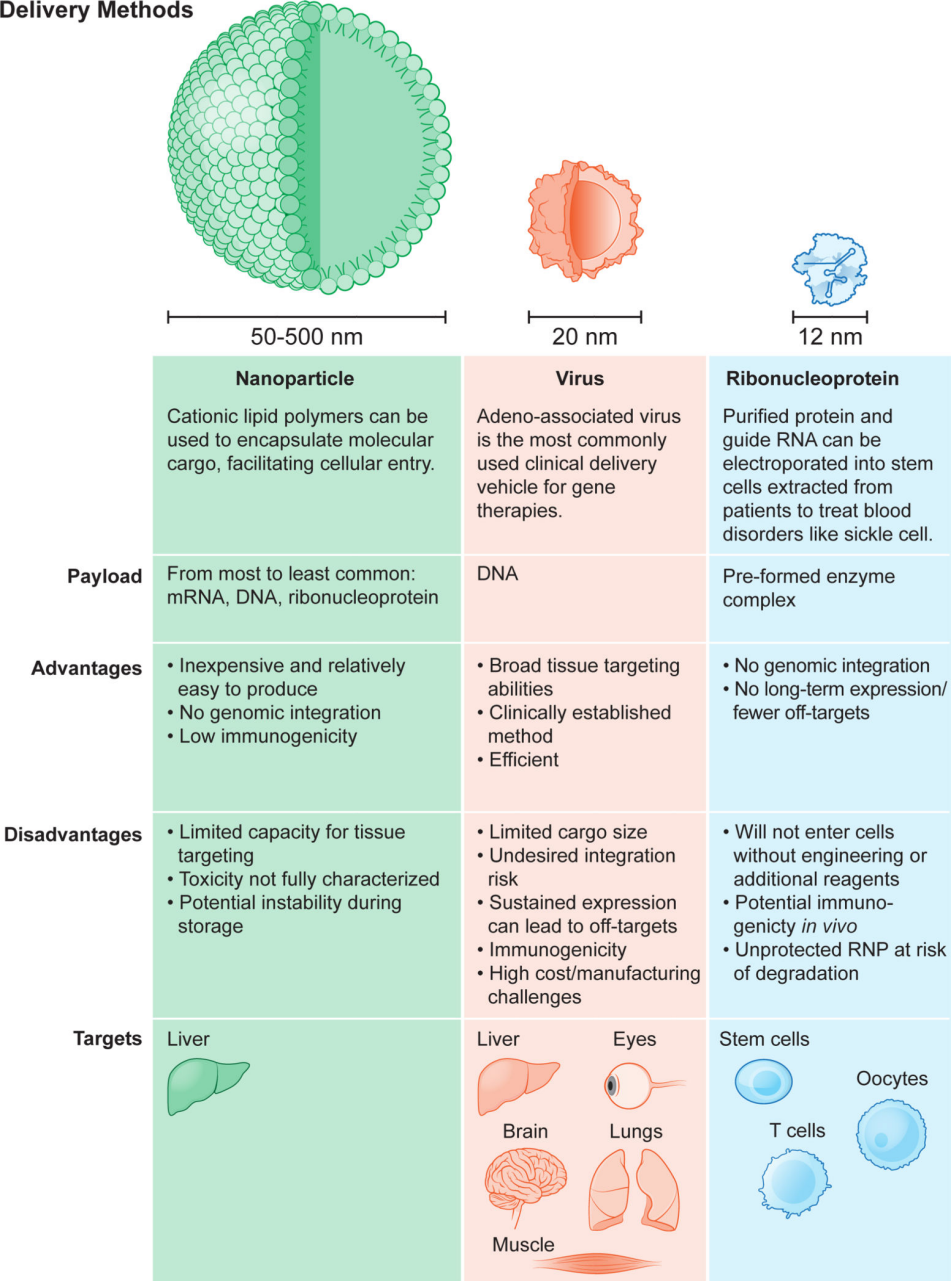


For any of these genome editing methods to be useful clinically, the CRISPR-Cas enzymes, associated guide RNAs and any DNA repair templates must make their way into the cells in need of genetic repair. To produce a functional genome editing complex, Cas9 and sgRNA can be introduced to cells in target organs in formats including DNA/DNA, mRNA/sgRNA, or protein/sgRNA, respectively. All three formats are currently, or shortly to be, used in the clinic, using viral vectors, nanoparticles and electroporation of protein-RNA complexes, and each has distinct benefits and limitations (Fig. 4). The currently favored form of *ex vivo* delivery to primary cells is electroporation of Cas9 as a preformed protein-RNA (ribonucleoprotein, RNP) complex^44,56^. *In vivo* delivery, which is much more challenging, is currently conducted using viral vectors (typically adeno-associated virus, AAV) or lipid nanoparticles bearing Cas9 mRNA and an sgRNA. The difficulty of ensuring efficient, targeted delivery into desired cells in the body currently limits the clinical opportunities of *in vivo* genome editing, although this is an area of increasing research and development.

Viral delivery vehicles, including lentivirus, adenovirus and adeno-associated virus (AAV), offer advantages of efficiency and tissue selectivity (Fig. 4). AAV is attractive due to its reduced risk of genomic integration, inherent tissue tropism and clinically manageable immunogenicity. In addition, long-term expression of trans-genes encoding Cas9 and sgRNA from the episomal viral genome could help boost genome editing efficiency in patients, such as those with Duchenne muscular dystrophy as discussed below^57^. Notably, the FDA has approved AAV for gene replacement therapy in spinal muscular atrophy and congenital blindness, and clinical trials are in progress^58^.

There are significant challenges to using AAV for therapeutic delivery of CRISPR-Cas components, however. First, the AAV genome can only encode ~4.7 kb of genetic cargo, less than other viral vectors and not much larger than the 4.2 kb length of the gene encoding *S. pyogenes* Cas9. As a result, in applications calling for corrective gene insertion, a second AAV vector encoding the sgRNA and/or a template sequence for homology-directed DNA repair must be used, reducing efficiency due to the need for cells to acquire both AAV vectors at once^59,60^. Smaller genome editing proteins, such as the *S. aureus* Cas9, *C. jejuni* Cas9 and newly identified CRISPR-Cas enzymes, may circumvent this issue^23,61–65^. Second, long-term expression of genome editing molecules may expose patients to undesired off-target editing or immune reactions^66,67^. Third, the production of AAV at scale and the employment of good manufacturing process (GMP) methods at affordable cost for clinical use remains a formidable challenge^68–70^.

Nanoparticles offer an alternative to viral-based delivery of Cas9 and sgRNAs and are suitable for delivering genome editing components in the form of DNA, mRNA, or ribonucleoprotein (RNP) (Fig. 4). For example, lipid-mediated nanoparticle (LNP) delivery has been used to transport CRISPR-Cas components in the form of either mRNA/sgRNA or preassembled RNPs into tissues^71–74^. When combined with the highly anionic sgRNA, the cationic Cas9 protein forms a stable RNP complex that has anionic properties suitable for encapsulation by cationic lipid nanoparticles, potentially enabling delivery into cells through endocytosis and macropinocytosis. Cationic lipid-based delivery is a relatively easy, low-cost process to deliver CRISPR components into cells^75^. This approach has been used for one-shot delivery of Cas9 RNPs into mice to achieve therapeutically useful levels of genome editing in the liver^55^. Disadvantages of this approach include significant toxicity of the LNPs^76^ and the sometimes undesired selectivity of cell-type specific uptake of the particles.

Inorganic nanoparticles are another type of delivery vehicle with advantages including tunable size and surface properties. Gold nanoparticles, in particular, are attractive materials for molecular delivery because of the intrinsic affinity of gold for sulfur, enabling functionalized molecules to be coupled to the gold particle surface. Gold nanoparticles were used originally for nucleic acid delivery by conjugating to thiol-linked DNA or RNA (reviewed in ref. 77). Cas9 protein-sgRNA complexes can be incorporated by assembly with DNA-linked particles^78,79^. Such assemblies, complexed with polymers capable of disrupting endosomes and including DNA templates for homology-directed repair, were found to promote correction of dystrophin gene mutations in mice^80^. Ongoing research continues to advance nanoparticle delivery technology, such as for endothelial cells that could enable access to lung and other organs^81^.

Strategies for nonviral cellular delivery of CRISPR-Cas components include electroporation, which involves pulsing cells with high-voltage currents that create transient nanometer-sized pores in the cell membrane. This process allows negatively-charged DNA or mRNA molecules or CRISPR-Cas RNPs to enter the cells. Although this method is a primary method of Cas9-sgRNA delivery to cells *ex vivo*, electroporation has also been used successfully for Cas9 delivery to animal zygotes^82,83^, and to introduce CRISPR-Cas constructs directly into mouse skeletal muscle, resulting in restoration of dystrophin gene expression^84^. Electroporation will likely be of limited utility for most *in vivo* genome editing applications due to impracticality.

Another non-viral delivery method is direct application of pre-assembled CRISPR-Cas RNPs, with or without chemical modifications to assist cell penetration, to cultured cells or organs. This delivery mode can reduce possible off-target mutations relative to delivering Cas9-encoding DNA or mRNA due to the short half-life of RNPs^76,85–87^. New strategies for direct delivery of CRISPR-Cas9 RNP complexes continue to emerge, including those using molecular engineering to enhance targeting of specific cell types^88^ and to increase the efficiency of cell penetration^89^.

Delivery remains perhaps the biggest bottleneck to somatic cell genome editing, a reality that has motivated increasing effort across different disciplines. New emerging strategies that may have substantial impact on clinical use of genome editing include advances in nanoparticle and cell-based delivery methods (reviewed in ref. 90) as well as approaches involving red blood cells (reviewed in ref. 91) and nanowires (see for example ref. 92).

## ACCURACY, PRECISION AND SAFETY OF GENOME EDITING

The clinical utility of genome editing depends fundamentally on accuracy and precision. Accuracy refers to the ratio of on-versus off-target genetic changes, whereas precision relates to the fraction of on-target edits that produce the desired genetic outcome. Inaccurate (off-target) genome editing occurs when CRISPR-induced DNA cleavage and repair happens at genomic locations not intended for modification, typically sites that are close in sequence to the intended editing site (reviewed in ref. 93). Imprecise genome editing results from different modes of DNA repair after on-target DNA cleavage, such as a mixture of non-homologous end joining and homology-directed recombination events that produce different sequences at the desired editing location in different cells. In addition, large deletions and complex genomic rearrangements have been observed after genome editing in mouse embryonic cells, hematopoietic progenitors and human immortalized epithelial cells^94–96^. Although these events occur at low frequency, they could be significant in a clinical setting if rare translocations led to cancer^97–99^. Careful testing will be required to detect and monitor both the accuracy and precision of genome editing in clinical settings and ultimately to reduce or eliminate undesired events by controlling target site recognition and DNA repair outcomes. The National Institute of Standards and Technology (NIST) manages a scientific consortium aimed at measuring and standardizing such outcomes as genome editing technology advances^100^.

The risks intrinsic to DNA cleavage-induced genome editing have spurred development of CRISPR-Cas9-mediated genome regulation or editing methods that do not involve double-stranded DNA cutting. CRISPR interference (CRISPRi) and CRISPR activation (CRISPRa) employ catalytically deactivated forms of Cas9 (dCas9) that are fused to transcriptional repressors or activators^29,101^. Similarly, CRISPR-Cas9-mediated epigenetic modification to control gene expression is also under development^102^. An alternative approach is to use CRISPR-Cas9 coupled to DNA editing enzymes that catalyze targeted A-to-G or C-to-T genomic sequence changes without inducing a break in DNA, potentially reversing pathogenic single-nucleotide changes or disabling genes via the introduction of a stop codon^25,26^. CRISPR-Cas9 can also be linked to reverse transcriptase and deployed for targeted template-directed sequence alterations^103^. All of these strategies, though elegant in principle, involve large chimeric proteins that are pose additional challenges of delivery into primary cells or animals. The specificity of action both at the target, as well as genome-wide, remains an area of active investigation. Issues of delivery, potency, and specificity of CRISPRi, CRISPRa and CRISPR-mediated base editing and prime editing will need to be thoroughly addressed before they are ready for clinical use.

Other factors affecting clinical applications of genome editing include the immunogenicity of bacterially-derived editing proteins, the potential for pre-existing antibodies against CRISPR components to cause inflammation and the unknown long-term safety and stability of genome editing outcomes. Immunogenicity of CRISPR-Cas proteins could be managed by high-efficiency one-time editing treatments and by using different editing enzymes. Pre-existing Cas9 antibodies and reactive T-cells have been detected in humans exposed to pathogenic bacteria harboring CRISPR systems, although it is unknown whether these are present at concentrations sufficient to trigger an immune response to the genome editing enzymes^66,104^. Notably, genome editing therapies that involve *ex vivo* editing, such as for sickle cell disease, are not as affected by either immunogenicity or pre-existing CRISPR-Cas antibodies, with residual Cas9 protein in the *ex vivo* edited cells being a manageable issue. The potential for inadvertent selection of genome-edited cells with undesired genetic changes came to light with the observation that selection for inactivation of the p53 pathway, which is associated with rapid cell growth and cancer, can occur during laboratory experiments on cells that are not used clinically^105,106^. Subsequent experiments showed that p53 inactivation can be controlled or avoided through protocol optimization^47,107^. As for long-term safety and efficacy of genome-edited cells *in vivo*, much remains to be determined. However, the recent report of a single HIV-positive patient who received CRISPR-Cas9-edited hematopoietic progenitor cells showed that although the number of edited cells was too low to mitigate HIV infection, no adverse outcome was detected over 19 months after transplantation of the edited cells^108^. Together, these findings suggest that there are, at present, no known insurmountable hurdles to the eventual development of safe and effective clinical applications of genome editing in humans.

## THERAPEUTIC GENOME EDITING

The clinical potential of genome editing exemplified by applications in sickle cell disease, muscular dystrophy and other monogenetic disorders could be stymied by extreme pricing of such next-generation therapeutics. Although CRISPR technology itself is a democratizing tool for scientists, extension of its broad utility in biomedicine requires addressing the costs of development, personalization for individual patients and the intrinsic difference between a chronic disease treatment versus a one-and-done cure (reviewed in ref. 103).

Current clinical trials using the CRISPR platform aim to improve chimeric antigen receptor (CAR) T-cell effectiveness, treat sickle cell disease and other inherited blood disorders, and stop or reverse eye disease^109^. In addition, clinical trials to use genome editing in degenerative diseases including muscular dystrophy patients are on the horizon. For sickle cell disease, the uniform nature of the underlying genetic defect lends itself to correction by a standardized CRISPR modality that could be used in many if not most patients. This simplifies clinical testing but also makes the need to address patient cost and access more acute, given that the ~100,000 US patients and millions more in African and Asian countries will be candidates for treatment.

For muscular dystrophy, the genetic diversity among patients lends itself to personalization that is an inherent strength of the CRISPR genome editing platform, yet also complicates clinical testing strategies. In addition, progressive diseases like muscular dystrophy require early treatment to be most effective, raising questions about coupling diagnosis and treatment. Beyond these examples, many rare genetic disorders will be treatable in principle if a streamlined strategy for CRISPR therapeutic development can be implemented^103^. With its potential to address unmet medical needs, clinical use of genome editing will ideally spur changes to regulatory guidelines and cost reimbursement structures that will benefit the field more broadly as these therapies continue to advance.

Notably, all of the genome editing therapeutics under development aim to treat patients through somatic cell modification. These treatments are designed to affect only the individual who receives the treatment, reflecting the traditional approach to disease mitigation. However, genome editing offers the potential to correct disease-causing mutations in the germline, which would introduce genetic changes that would be passed on to future generations. The scientific and societal challenges associated with human germline editing are distinct from somatic cell editing and are discussed in the next section.

## HERITABLE GENOME EDITING

Human germline genome editing can introduce heritable genetic changes in eggs, sperm or embryos. Germline genome editing is already in widespread use in animals and plants and has been employed in human embryos for research purposes. A report of alleged use of human embryo editing resulting in the birth of twin baby girls with edited genomes has focused global attention on an application of genome editing that must be rigorously regulated, as underscored by international scientific organizations.

Human germline editing differs from somatic cell editing because it results in genetic changes that are heritable if the edited cells are used to initiate a pregnancy (Fig. 5). Germline editing has been used for years in animals, including mice, rats, monkeys and many others, and experiments show that it can be done in both nonviable or viable human embryos as well^110–113^. Although none of the published work involves implantation of the edited embryos to initiate a pregnancy, such work was reported at a conference on human genome editing in November 2018, leading to international condemnation in light of clear violations of ethical and scientific guidelines.

This work and the accompanying discussion around human germline editing have raised important questions that affect the future direction of the science as well as the societal and ethical issues that accompany any such applications. First, research using CRISPR-Cas9 in human embryos has challenged current understanding of DNA repair mechanisms and developmental pathways that occur in these cells. A report of inaccurate CRISPR-Cas9-based genome editing in non-viable human embryos^110^ was not substantiated by later publications, but the mechanism by which double-stranded DNA breaks are repaired in early human embryos remains under debate. Some results were interpreted to indicate repair of a CRISPR-Cas9targeted gene allele by homology-directed repair with the cell’s other allele as the donor template^114^. Other scientists argued that such repair would be impossible given the apparent physical separation of sister chromatids early in embryogenesis, and suggested the data could also be consistent with large deletions in the embryo genomes^94,115^. Resolving this fundamental question will require further experiments. Human embryo editing has also begun to reveal differences in the genetics of early development in mice versus humans^111^, underscoring the potential value of research that will be enabled by precision genome modification.

A second question raised by applications of genome editing in human embryos concerns the appropriate professional and societal response. Organizations including the National Academy of Sciences, the National Academy of Medicine, the Royal Society and their equivalents in other countries have sponsored meetings and reports, as have professional societies including the American Society of Human Genetics (ASHG)^116^, UK Association of Genetic Nurses and Counsellors, Canadian Association of Genetic Counsellors, International Genetic Epidemiology Society, US National Society of Genetic Counselors, American Society for Reproductive Medicine, Asia Pacific Society of Human Genetics, British Society for Genetic Medicine, Human Genetics Society of Australasia, Professional Society of Genetic Counselors in Asia, and Southern African Society for Human Genetics. Key points on which these groups agree: (1) At this time, given the nature and number of unanswered scientific, ethical, and policy questions, it is inappropriate to perform germline genome editing that culminates in human pregnancy; (2) *in vitro* germline genome editing on human embryos and gametes should be allowed, with appropriate oversight and consent from donors, to facilitate research on the possible future clinical applications of gene editing, and there should be no prohibition on public funding of this research; (3) future clinical application of human germline genome editing should not proceed unless, at a minimum, there is (a) a compelling medical rationale, (b) an evidence base that supports its clinical use, (c) an ethical justification, and (d) a transparent public process to solicit and incorporate stakeholder input.

The third question raised by applications of CRISPR-Cas9 in human embryos is how to move the technology forward while ensuring responsible use. At the time of this writing, international commissions convened by the World Health Organization (WHO) and by the US National Academy of Sciences and National Academy of Medicine, together with the Royal Society, are drafting detailed requirements for any potential future clinical use. Medical needs must be defined so that risks versus possible benefits can be evaluated. Most importantly, procedures by which patients could be informed about the technology, its risks and a process for monitoring health outcomes must be determined.

## OUTLOOK

Therapeutic genome editing will be realized, at least for some diseases, over the coming 5–10 years. This profound opportunity to change healthcare for many people requires scientists, clinicians and bioethicists to work with healthcare economists and regulators to ensure safe, effective and affordable outcomes. The potential impact on patients is too important to wait.

## Figures and Tables

**Fig. 5:**